# Cost-effectiveness analysis of Shexiang Baoxin Pill (MUSKARDIA) as the add-on treatment to standard therapy for stable coronary artery disease in China

**DOI:** 10.1371/journal.pone.0299236

**Published:** 2024-03-01

**Authors:** Jie Pan, Ping-da Ping, Wei Wang, Jia-meng Zhou, Wen-tao Zhu

**Affiliations:** 1 School of Traditional Chinese Medicine, Beijing University of Chinese Medicine, Beijing, China; 2 School of Management, Beijing University of Chinese Medicine, Beijing, China; Harvard University T H Chan School of Public Health, UNITED STATES

## Abstract

**Background:**

Recent evidence indicates that Shexiang Baoxin Pill (MUSKARDIA), as an add-on treatment to standard therapy for stable coronary artery disease (CAD), is effective. Nevertheless, the cost-effectiveness of introducing the Shexiang Baoxin Pill (Abbreviation SBP) to the current standard treatment for patients with CAD in China remains unknown.

**Objective:**

The objective of this study was to assess the cost-effectiveness of introducing SBP into the current standard treatment in China for patients with CAD.

**Method:**

The effects of two treatment strategies—the SBP group (SBP combined with standard therapy) and the standard therapy group (placebo combined with standard therapy)—were simulated using a long-term Markov model. The simulation subjects might experience non-fatal MI and/or stroke or vascular or non-vascular death events. The study parameters were primarily derived from the MUSKARDIA trial, which was a multicenter, double-blind, placebo-controlled phase IV randomized clinical trial. Furthermore, age-related change, event costs, and event utilities were drawn from publicly available sources. Both costs and health outcomes were discounted at 5.0% per annum. One-way and probabilistic sensitivity analyses were conducted to verify the robustness of the model. Based on the MUSKARDIA trial results, the risk with the events of major adverse cardiovascular events (MACE) was decreased (*P* < 0.05) in the female subgroup treated with SBP therapy compared with standard therapy. Consequently, a scenario analysis based on subgroups of Chinese females was conducted for this study. Incremental cost-effectiveness ratios (ICERs) were assessed for each strategy for costs per quality-adjusted life-year (QALY) saved.

**Results:**

After 30 years of simulation, the SBP group has added 0.32 QALYs, and the cost has been saved 841.00 CNY. Compared with the standard therapy, the ICER for the SBP therapy was -2628.13 CNY per QALY. Scenario analyses of Chinese females showed that, after 30 years of simulation, the SBP therapy has been increased by 0.82 QALYs, and the cost has been reduced by 19474.00 CNY. Compared with the standard therapy, the ICER for the SBP therapy was -26569.51 CNY per QALY. Similar results were obtained in various extensive sensitivity analyses.

**Conclusions:**

This is the first study to evaluate the cost-effectiveness of SBP in the treatment of CAD. In conclusion, SBP as an add-on treatment to standard therapy appears to be a cost-effective strategy for CAD in Chinese patients.

## Introduction

Cardiovascular disease (CVD) is the leading cause of death, disability, and morbidity globally, most notably in China. The incidence of cardiovascular disease in China is increasing. The 2020 report on cardiovascular health and disease in China estimated 330 million patients with CVD and 11.39 million with coronary heart disease [1]. Data from the 2020 Chinese Health Statistical Yearbook showed that the mortality rate for coronary heart disease in 2019 was 121.59 per 100,000 urban residents and 130.14 per 100,000 rural residents, which is higher in rural areas than in urban areas [2]. The economic burden of cardiovascular disease for residents and society is increasing daily, and it has become a major public health issue.

Primary prevention of CAD focuses on risk factors including hypertension, hyperlipidemia, diabetes, etc. The secondary prevention of CAD is to prevent exacerbation and reduce the associated mortality. Commonly used drugs include β-blockers, calcium channel blockers (CCBs), angiotensin-converting enzyme inhibitors (ACEIs)/angiotensin receptor blockers (ARBs), diuretics, and statins [3]. Although the above treatment improved the prognosis of patients, it did not reduce mortality [4, 5]. Beta-blockers may increase the risk of cardiogenic shock and thrombolytic agents can relieve symptoms but may also lead to stroke and extensive bleeding [6, 7]. In addition, due to tolerability problems caused by gastrointestinal reactions, exacerbated respiratory disease, gout, or hyper-uricemia, many patients with CAD in China cannot get the best treatment [8, 9]. Therefore, new alternative therapies are necessary to address tolerability and reduce residual cardiovascular risk [10, 11].

Traditional Chinese medicine (TCM) can be a potential adjunctive treatment and has been used for many years to treat CAD [12] SBP preserving heart bolus is the most commonly used fragrant and warm medicine of Chinese proprietary medicine at present. Its major function is fragrant wentong, yiqiqiangxin. It is used for chest pain caused by qi stagnation and blood stasis. The symptoms are precordial pain and fixed, myocardial ischemia caused by angina pectoris; myocardial infarction sees the above syndromes [13, 14]. It is composed of seven medicinal materials, including Moschus, total gingenoside ginseng root, Styrax, Cinnamomi Cortex, Bufonis Venenum, Bovis Calculus Artifactus, and Borneolum Syntheticum. Each ingredient contains a large number of chemical compounds. Like all TCM formulae, SBP is a multi-component and multi-target agent from the molecular perspective. As a result, its mode of action is most likely to be illustrated by network pharmacology-based approaches. [15–18]. Modern pharmacological studies suggest that SBP can protect the myocardium and improve cell mitochondrial function [19]. It can promote angiogenesis has anti-angiosclerosis effects and inhibits lipid accumulation [20, 21]. Numerous clinical pieces of evidence have fully proven the effectiveness and safety of MUSKADRIA [14, 22, 23], and many guidelines in China recommend its use in cardiovascular diseases [24–27]. Prior evidence indicates that SBP has a therapeutic effect on all types of chronic coronary syndromes (CCS) and acute coronary syndromes (ACS) [22], and long-term application can improve the symptoms of patients with coronary heart disease and reduce cardiovascular events [14, 28]. The findings were reported by a systematic review and meta-analysis of 19 eligible trials concluding that the combination of SBP with conventional treatment trial groups could significantly reduce the incidence of MACE, enhance left ventricular ejection fraction (LVEF), and lessen N-terminal pro-B-type natriuretic peptide (NT-pro-BNP) levels [29]. Similarly, the results from a systematic review and network meta-analysis of 179 eligible trials suggested that SBP combined with Western medicine is presumably the optimal treatment prescription for both clinically effective and cardiovascular event rates [30]. The present evidence confirmed the relative long-term safety of SBP, which does not affect liver and kidney function or increase the risk of bleeding [14, 23, 28, 31, 32].

In 2018, among the average hospitalization costs for cardiovascular diseases, the average hospitalization cost for ischemic heart disease was 13 083.90 Chinese yuan (CNY), (the cost for) myocardial infarction was 28,879.30 CNY; (the cost for) ischemic stroke was 9,409.64 CNY, intracranial hemorrhage 18,863.63 CNY. Excluding the impact of price factors, since 2004, the average annual growth rate of sub-hospitalization costs for acute myocardial infarction, ischemic stroke, and intracranial hemorrhage has been 6.09%, 1.26%, and 4.73%, respectively. Both early revascularization and late chronic disease management impose a tremendous economic burden on limited healthcare resources [2]. Several studies have reported the findings of pharmaco-economic assessments of aspirin, statins, etc., used for primary or secondary prevention in patients with cardiovascular disease [33–36]. As an efficient add-on therapy, there have been no studies on the cost-effectiveness of SBP for CAD in Chinese populations. In this study, we aimed to examine the cost-effectiveness of SBP use as an add-on treatment for CAD in Chinese patients, which might help public policymakers consider more economical strategies to be applied.

## Methods

### Study population

This hypothetical cohort is similar to the MUSKARDIA trial population [14]. The MUSKARDIA trial is a randomized, double-blinded, placebo-controlled, phase IV trial conducted at 97 sites in China (chictr.org.cn, ChiCTR-TRC-12003513), which included 2674 patients (1 case was rejected due to data quality) and consisted of 1342 in the SBP group and 1331 in the placebo group [14]. The trial was approved by the ethics committee of each participating center. Each subject provided written informed consent before recruitment [14].

### Model structure

A Markov state-transition model was developed to estimate the cost-effectiveness of SBP (two pills, three times daily, 135 mg in total) combined with standard therapy in a hypothetical cohort of over 65-year-old CAD patients. The population age was similar to the mean age (63.8-year-old CAD) of patients in the MUSKARDIA trial [14]. The primary characteristics of patients simulated in the present study were consistent with those who participated in the MUSKARDIA trial. Presented with stable ischemic myocardial symptoms for at least 1 month and had at least one of the following events: (1) history of acute myocardial infarction (MI) over 6 months; (2) history of percutaneous coronary intervention (PCI) or coronary artery bypass graft surgery (CABG) over 6 months; (3) epicardial coronary stenosis of ≥ 50% in at least one major branch as indicated by coronary computed tomography (CT) angiography or coronary angiography.

**Fig 1:**
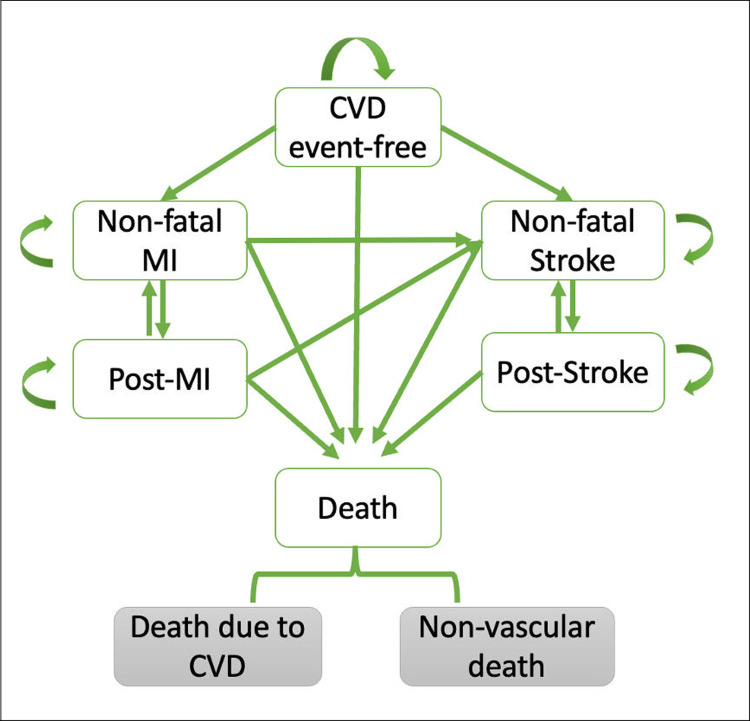
Markov state-transition model diagram. All model subjects entered the model in the initial state as CVD event-free. Within the long-term simulation, patients could move in the direction of any arrow in each cycle. CVD: cardiovascular disease; MI: myocardial infarction.

The model structure is conceptualized in Fig 1. Six model situations were defined with an initial state CVD event-free:

- CVD event-free;
- non-fatal MI;
- post-MI;
- non-fatal stroke;
- post-stroke;
- vascular death (MI or stroke)/non-vascular death.

This model has been used in recent studies [33–36]. The model cycle length was 1 year, and half-corrections were applied. The time horizon was 30 years to cover most of the Chinese population. During each 1-year cycle, patients may experience a new MI, a new stroke, die, or be in a post-event state, but not both MI and stroke within the same year. Patients who experienced non-fatal MI or non-fatal stroke would transit to the post-MI or post-stroke state in the following year. Patients who have experienced a non-fatal stroke will no longer be able to transition to a new non-fatal myocardial infarction state, as this would allow the stroke patient to transition to a health state that improves the quality of life and reduces associated costs.

The model evaluated QALYs and the total costs of the SBP and standard therapy, respectively. The primary outcome was the ICER, equal to the cost per QALY saved. All costs were estimated in Chinese yuan (CNY). Granting to the recommendation of the China Guidelines for Pharmacoeconomic Evaluations, cost-effectiveness was determined assuming a threshold of three times per capita gross domestic product (GDP) (80,976.00 CNY×3 = 242,928.00 CNY in 2021) [37, 38]. TreeAge 2012 software program (TreeAge Software LLC, Williamstown, MA, USA) was used for statistical analyses.

### Scenario analyses

The subgroup analyses of the MUSKARDIA trial results showed that females who received SBP therapy had a lower risk of MACE events (*RR* = 0.3, *95%CI* 0.08 ~ 0.99, *P* = 0.0474) [14]. Therefore, a scenario analysis was conducted to evaluate the cost-effectiveness of SBP for Chinese females with CAD.

### Clinical inputs

Clinical inputs are shown in Table 1. The relative risk of non-fatal MI and non-fatal stroke in CAD patients with the use of SBP was estimated from the pooled data reported in the MUSKARDIA trial [14], which examined the clinical outcomes of SBP in 2674 Chinese patients with a follow-up of 2 years. Age-specific all-cause mortality rates and age-specific CVD-related mortality estimates were derived from the Sixth Census and the Health Statistics Yearbook 2020, respectively [2, 39]. The annual event rates of non-fatal stroke were results from a nationwide population-based survey of 480 687 adults [38]. Stroke recurrence rates were derived from a 9-year community study of 2.5 million samples, and the probability was calculated based on the five-year recurrence rate after the first stroke [40]. According to the China Stroke Prevention and Control Report 2020 [41], the stroke recurrence rate ranged from 5.59% to 11.65%, and this range was used for sensitivity analysis. The annual event rates and recurrence of non-fatal MI were calculated based on data from the China Patient-Centered Evaluative Assessment of Cardiac Events Prospective Study of acute myocardial infarction [42, 43]. The aforementioned transition probabilities of non-fatal MI were not an age-dependent parameter and need to be periodically adjusted according to age-related changes in mortality recorded for China-specific death rates [33]. Based on the 2020 Health Statistics Yearbook, for example, the mortality associated with MI was estimated at 0.11% and 0.37% in 65-69-year and 70-74-year age groups, respectively. With increasing age, death rates increase by a factor of 1.78 [2]. Mortality risk-increasing factors were applied to MI transition probabilities per 5 cycles until 80 years old, and then the simulation was continued with the same transition probability. The non-vascular death risks were recalculated after the exclusion of CVD death from the all-cause mortality to represent the natural decrease in the Chinese population [33].

**Table 1:** Key inputs in the model.

Parameter	Base-case value	Range	Distribution	Reference
Event rate				
All-case mortality				
65 to 69	0.0117	Age dependent	-	[39]
70 to 74	0.0212	Age dependent	-	[39]
75 to 79	0.0372	Age dependent	-	[39]
80 to 84	0.0661	Age dependent	-	[39]
≥85	0.1075	Age dependent	-	[39]
Death due to MI				
65 to 69	0.0011	Age dependent	-	[2]
70 to 74	0.0020	Age dependent	-	[2]
75 to 79	0.0038	Age dependent	-	[2]
80 to 84	0.0069	Age dependent	-	[2]
≥85	0.0209	Age dependent	-	[2]
Death due to stroke				
65 to 69	0.0007	Age dependent	-	[2]
70 to 74	0.0015	Age dependent	-	[2]
75 to 79	0.0033	Age dependent	-	[2]
80 to 84	0.0060	Age dependent	-	[2]
≥85	0.0167	Age dependent	-	[2]
Non-fatal stroke				
60 to 69	0.0082	Age dependent	-	[38]
70 to 79	0.0135	Age dependent	-	[38]
≥80	0.0210	Age dependent	-	[38]
Stroke recurrence	0.1001	0.0559 to 0.1165	β	[38, 40, 41]
Non-fatal MI	0.0170	Age dependent	-	[42]
MI recurrence	0.0410	0.0304 to 0.0521	β	[43]
RR of MUSKARDIA therapy versus no use of MUSKARDIA				
Non-fatal MI	0.6000	0.3200 to 1.3000	Lognormal	[14]
Non-fatal stroke	0.7000	0.3100 to 1.7700	Lognormal	[14]
Utility Inputs				
CVD event-free	0.8500	0.6900 to 1.0000	β	[14]
Non-fatal MI	0.6500	0.6300 to 1.0000	β	[14, 48]
Post-MI	0.6500	0.6300 to 1.0000	β	[14, 48]
Non-fatal stroke	0.5400	0.2200 to 0.8000	β	[14, 46, 47]
Post-stroke	0.5400	0.5300 to 0.9000	β	[14, 46, 47]
Cost Input*, CNY				
Non-fatal MI	35170.1500	16080.1600 to 36449.7100	Gamma	[2]
Post-MI	9876.6600	7407.4900 to 9259.3700	Gamma	[49]
Non-fatal stroke	11355.2600	5631.8800 to 16800.6000	Gamma	[2]
Post-stroke	10017.0100	7512.7600 to 12521.2600	Gamma	[49]
Death due to MI	23441.4600	17581.0900 to 29301.8200	Gamma	[49]
Death due to stroke	29126.4300	21844.8200 to 36408.0300	Gamma	[49]
Annual cost of drugs, CNY				
MUSKARDIA	1551.2500	1299.4000 to 1664.4000	Gamma	Calculate
Standard therapy	3788.7000	2036.7000 to 5540.7000	Gamma	Calculate

### Costs and utility

Seattle scale scores of patients in The MUSKARDIA trial were mapped to health utility scores [44]. The WOSCOPS clinical trial and its extended follow-up study suggested that each stroke, myocardial infarction, heart failure, and other coronary heart disease can reduce the quality of life. The quality of life will be reduced by 37.1% after stroke and by 24% after myocardial infarction [45].

Direct medical costs were included in the present study. The price data of SBP came from the implementation price of Mi Nei net (https://www.menet.com.cn) in the past year (2022 year), with a total of 8 winning bid data. The average daily cost of SBP is 4.25 CNY, and the average daily cost range is 3.56 to 4.56 CNY. In the MUSKARDIA trial, the standard treatment drugs for chronic stable coronary heart disease were not specified. The patients’ medication and standard drug adjustment are based on the disease conditions and doctor’s orders, which are more in line with the real-world medication situation. Therefore, the average daily cost of standard treatment is mainly calculated based on the medication records of patients in the MUSKARDIA trial. The costs of nonfatal MI and nonfatal stroke were derived from the 2020 China Health Statistic Yearbook [2], which were the one-time direct medical costs for the management of MI and stroke events. The costs of Post-MI, Post-stroke, death due to MI, and death due to stroke were derived from the Li YG et al and raised 15% to reflect some unmeasurable costs except medication costs [49]. All costs (including events and medication), as well as health outcomes, were discounted by 5% annually according to Chinese guideline recommendations [37].

### Sensitivity analysis

Sensitivity analyses were conducted to examine the robustness of the model results. One-way sensitivity analysis over variable ranges (95% confidence interval (CI) or ±25% of base-case values) was performed to examine the impact of each variable on the results. The annual discount rate of costs and utilities in one-way sensitivity analysis ranged from 0% to 8%, and the time horizon ranged from 10 years to 30 years. Beta and gamma distributions were assigned to event probabilities, utilities, and cost estimates, respectively in the probabilistic sensitivity analysis. The probabilistic sensitivity analysis was performed by Monte Carlo simulations set to simulate 2,000 times.

## Results

### Main results

The base-case results are shown in Table 2. SBP therapy was less costly (103889.00 CNY versus 104730.00 CNY) with higher QALYs gained (9.12 versus 8.80 QALYs) compared to placebo. SBP therapy was a cost-effective option with an ICER of -2628.13 CNY/QALY.

**Table 2:** Total costs, QALY and ICER in two strategies for modeling effectiveness.

Strategy	Cost(CNY)	Effectiveness (QALYs)	Incremental		
			Cost	QALYs	Cost/QALYs
Base-case results					
The placebo treatment	104730.00	8.80			
The MUSKARDIA treatment	103889.00	9.12	-841.00	0.32	-2628.13
Scenario analysis results					
The placebo treatment	104730.00	8.80			
The MUSKARDIA treatment	82943.00	9.62	-21787.00	0.82	-26569.51

Note: QALY: quality-adjusted life year; CNY: Renminbi; The placebo treatment: placebo combined with standard treatment; The MUSKARDIA treatment: MUSKARDIA combined with standard treatment.

### Scenario analyses

The scenario analysis results are shown in Table 2. After 30 years of simulation analysis in the female subgroup, the benefit of SBP was higher QALYs gained (9.62 versus 8.80 QALYs) compared to the placebo. SBP therapy was a cost-effective option with an ICER of -26569.51 CNY/QALY.

### Sensitivity analyses

**Fig 2:**
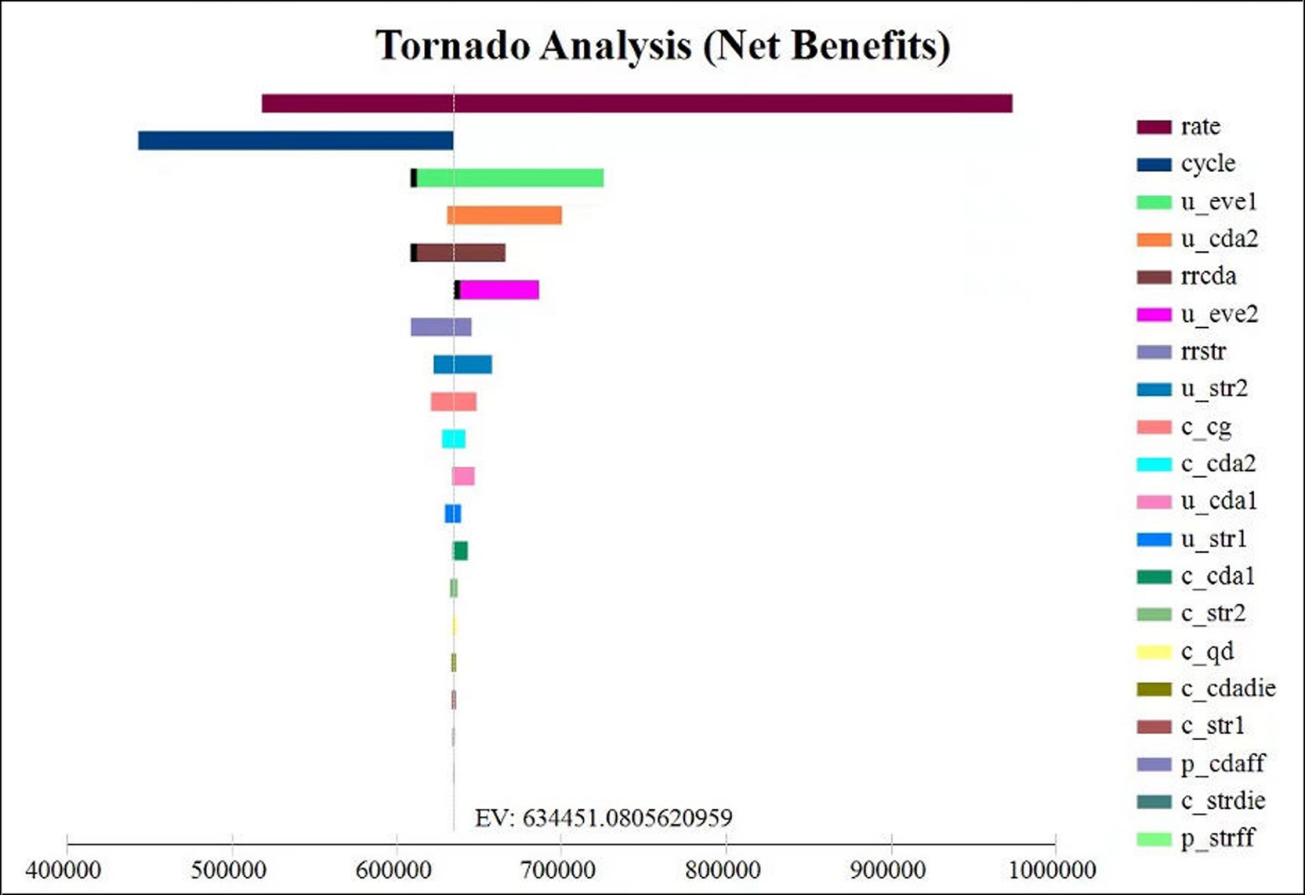
Tornado diagrams in one-way deterministic sensitivity analyses. EV: expected value; rate: discount rate; u_eve1 and u_eve2: utility of CVD event-free; rrcda: RR of non-fatal MI; rrstr: RR of non-fatal stroke; u_cda1: utility of non-fatal MI; u_cda2: utility of post-MI; u_str1: utility of non-fatal stroke; u_str2: utility of post-stroke; c_cda1: cost of non-fatal MI; c_cda2: cost of post-MI; c_str1: cost of non-fatal stroke; c_str2: cost of post-stroke; c_qd: Annual cost of Shexiang Baoxin Pill; c_cg: Annual cost of standard therapy; c_cdadie: cost of death due to MI; c_strdie: cost of death due to stroke; p_cdaff: MI recurrence rate; p_strff: stroke recurrence rate.

The one-way sensitive analysis results are shown in S1 Table and Fig 2. In one-way deterministic sensitivity analyses, the ICER was closely associated with the discount rate, cycle, and utility of post-MI. When the discount rate changes to 8%, the placebo becomes less costly (76894.33 versus 77444.28 CNY) with lower QALYs gained (7.13 versus 7.35 QLAYs) compared to SBP, SBP still was a cost-effective option with ICER 2426.75 CNY/QALY below the WTP threshold. Similarly, after those parameters were adjusted to the corresponding values, the SBP is still the cost-effective option with higher costs and higher QALYs gained compared to Placebo, the ICERs were below the WTP.

In particular, the main results are sensitive to the relative risk of nonfatal MI (0.6; range: 0.32–1.30). Placebo would become cost-effective when the relative risk of nonfatal MI exceeded 1.30. When the relative risk of nonfatal MI was lower than 1.30, SBP therapy gained higher QALYs at a higher cost with ICERs below the WTP threshold. The SBP therapy was shown to be cost-effective with a longer follow-up duration. Within the range of the parameters, the results showed robustness and the trend remained consistent.

**Fig 3:**
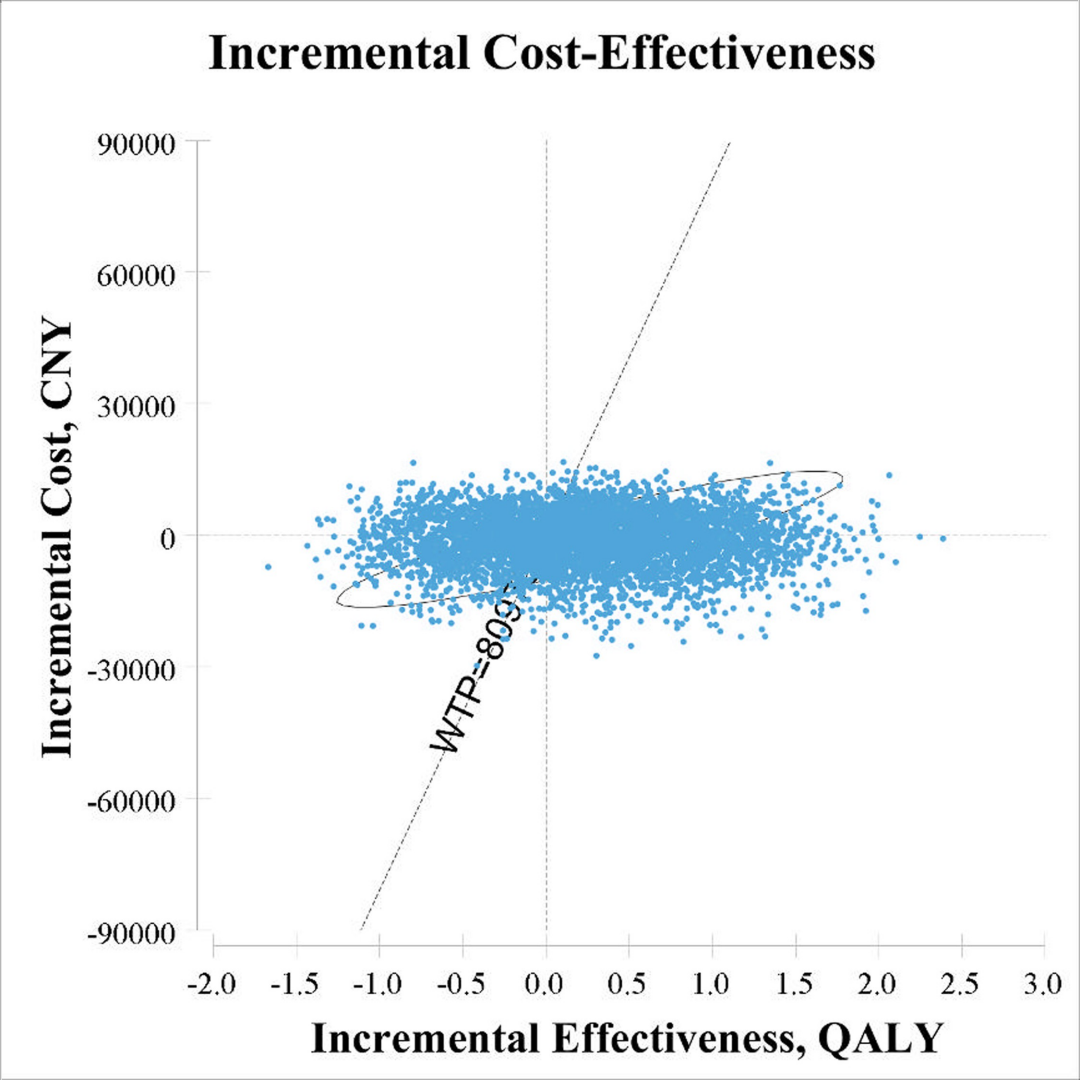
Monte Carlo simulation scatter plot in probabilistic sensitivity analyses. The ellipse shows the 95% confidence intervals, and the dotted line shows the willingness-to-pay threshold, with a slope of 80976 CNY per quality-adjusted life-year gained. CNY: Chinese yuan; QALY: quality-adjusted life-year; WTP: willingness-to-pay.

**Fig 4:**
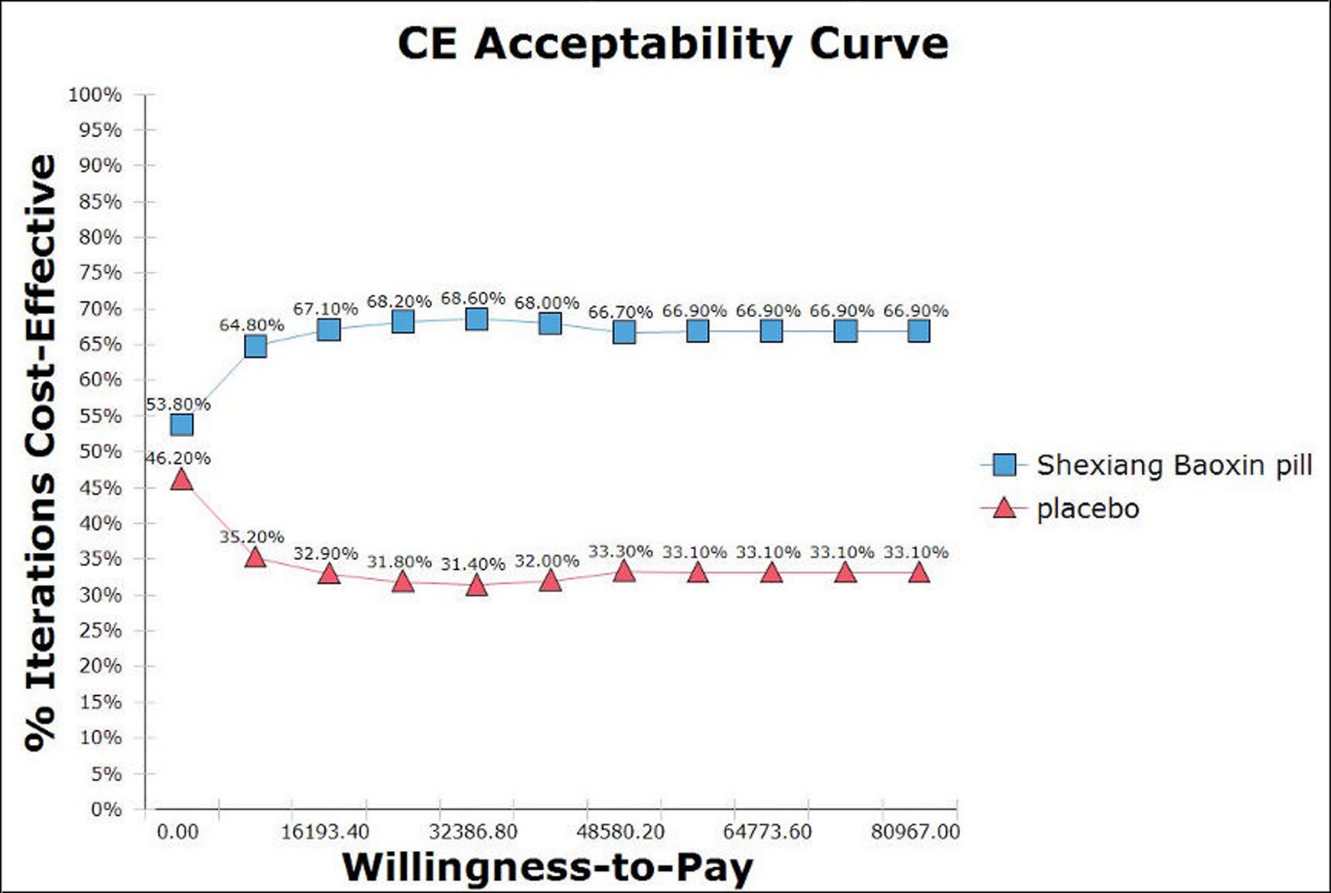
Cost-effectiveness acceptability curves in probabilistic sensitivity analyses.

The Monte Carlo simulation scatter plot (Fig 3) showed that 81.42% of the simulated population was willing to pay the threshold cost (8,0976 CNY/QALY) for the SBP therapy. A total of 15.25% of spots fell in the second quadrant, where SBP therapy was less effective and costly. These dominant spots may be the related relative risk of nonfatal MI according to one-way sensitivity. Using 1-fold GDP per capita as the WTP threshold (8,0976 CNY/QALY), the probability of SBP therapy and placebo being cost-effective were 66.90% and 33.10%, respectively (Fig 4).

## Discussion

To the best of our knowledge, this is the first analysis to examine the costs and health outcomes of SBP in Chinese patients with CAD. The present study indicated that SBP would be cost-effective as an add-on treatment for standard therapy in the general CAD population, which is mainly due to its higher efficacy resulting in fewer MACE events and ultimately less related mortality over time. The scenario analysis results showed that the female subgroup might obtain more benefits. One-way and probabilistic sensitivity analyses were conducted to verify the robustness of the model. Probabilistic sensitivity analysis further supported SBP therapy to be preferred in 66.90% of 2,000 times Monte Carlo simulations when using GDP per capita as the WTP threshold (8,0976 CNY/QALY).

Most parameters were adjusted without affecting the main results, namely, the SBP is still the cost-effective strategy, or the ICER is lower than the WTP and the added cost is worthwhile. The robustness of the main results was sensitive to the relative risk of nonfatal MI (0.6; range: 0.32–1.30). When the relative risk of nonfatal MI exceeded 1.30, SBP therapy was associated with a higher cost (spending 15,673.70 CNY more) and gained lower QALYs (gained 0.05 QALYs less). The current research supports that SBP has adjunctive effects on AMI via the mechanism of antioxidative stress [50]. Furthermore, the results of a recent meta-analysis showed that SBP combined with standard therapy had a lower relative risk of cardiovascular events compared with standard treatment of coronary heart disease alone (RR = 0.41; 95%CI: 0.35–0.47) [51]. As discussed above, a relative risk of nonfatal MI greater than 1.3 may not be robust on current evidence, and stronger evidence is required to justify it.

The sensitivity analysis is discussed more comprehensively in the study. Foremost, post-MI and post-stroke event costs are supported by the research on Chinese hospital data [49]. However, these data only include drug costs and do not contemplate the actual economic burden of the disease due to the higher disability rate of stroke and the costs of outpatient and adverse events after myocardial infarction. A study of secondary prophylaxis drugs in patients with acute coronary syndrome in Tianjin showed that the annual per capita all-cause direct medical costs of the total sample patients during the follow-up period of 12 months were 21,346 (2019-year data), and 88.7% of patients had at least one disease-specific outpatient record [52]. Another study on the economic burden and risk of chronic diseases in China also showed that patients with cardiovascular diseases had the highest overall economic burden of disease and economic risk, 8,954.29 (2014-year data) and 1.36, respectively [53]. Due to the lack of relevant research data support, the range of basic data and sensitivity analysis was adjusted to take into account the impact of the above factors on the economic burden of the disease. The results of the one-way sensitivity analysis are relatively robust, with the SBP therapy being the cost-effective strategy for most parameter values.

For the first time, the study analyzed the economics of using SBP in a subgroup of females with CAD. A population-based retrospective cohort study analyzed 201,921 patients with AMI, of whom 31.28% were female [54]. The study suggested that women have an increased risk of nonfatal stroke and heart failure. Women with AMI have poorer short- and long-term outcomes, during hospitalization to the 3-year follow-up. Women with acute myocardial infarction may be at higher risk of major adverse cardiac events. This study’s interesting finding is that SBP might have additional benefits for female patients in China, both in terms of efficacy and cost.

### Study limitations

The study still has some limitations. The first is that not all the costs were included. The costs of this study mainly considered the direct medical costs (mainly drug costs, outpatient and inpatient costs). It cannot fully reflect the mental pain, suffering, and social welfare losses caused by the disease to the patient. While this study enhances the robustness of the conclusions by broadening the sensitivity analysis range, the real-world economic burden on patients still needs to be supported by stronger cost data. Second, the cost and utility parameters used in scenario analysis for the female subgroup remain the same as the general population and may lead to some bias. Studies on population health utility values have shown that there are differences in health utility values between men and women, and also with the overall population sample. However, due to the lack of directly measured health utility values, it is unclear whether the health utility values of women in this study were high or low compared to the overall population. Female subgroup analysis results showed lower risk in clinical studies [14]. Therefore, the application of SBP may produce more beneficial effects in improving the quality of life. Finally, the utility values in the study parameters were mainly derived from the Seattle-scale fraction mapping in the SBP trial, so there might be some bias [40]. We determined the range of broader sensitivity analysis by comprehensively searching for health utility values, thereby reducing the impact of bias on the conclusions. The results of the sensitivity analysis have shown the robustness of the research conclusions at different values. In the future, patient-reported outcomes should be evaluated to obtain health utility values based on Chinese preferences.

## Conclusions

This is the first study to evaluate the cost-effectiveness of SBP in the treatment of CAD. In conclusion, SBP as an add-on treatment to standard therapy appears to be a cost-effective strategy for CAD in Chinese patients.

## Supporting information

S1 Table One-way sensitive analysis results. (DOCX)
