# Supplementary material for: Cost-effectiveness analysis of Shexiang Baoxin Pill (MUSKARDIA) as the add-on treatment to standard therapy for stable coronary artery disease in China
Source: PLoS One. 2024 Mar 1;19(3):e0299236. doi: 10.1371/journal.pone.0299236 (PMC10906875; doi:10.1371/journal.pone.0299236)
Supplement: S1 Table — (DOCX) [file pone.0299236.s001.docx]

**S1 Table. One-way sensitive analysis results**

| Parameter | Value | Group | Cost, CNY | | QALYs | | ICER, CNY |
| --- | --- | --- | --- | --- | --- | --- | --- |
|  |  |  | Total | Incremental | Total | Incremental |  |
| rate | 0.00 | placebo | 199253.33 |  | 13.81 |  |  |
|  | 0.00 | MUSKARDIA | 193329.43 | -5923.90 | 14.41 | 0.60 | -9948.44 |
|  | 0.04 | placebo | 117486.50 |  | 9.53 |  |  |
|  | 0.04 | MUSKARDIA | 115980.56 | -1505.94 | 9.88 | 0.35 | -4263.82 |
|  | 0.08 | placebo | 76894.33 |  | 7.13 |  |  |
|  | 0.08 | MUSKARDIA | 77444.28 | 549.95 | 7.35 | 0.23 | 2426.75 |
| u_cda1 | 0.63 | placebo | 104730.20 |  | 8.79 |  |  |
|  | 0.63 | MUSKARDIA | 103888.65 | -841.55 | 9.11 | 0.32 | -2655.58 |
|  | 0.81 | placebo | 104730.20 |  | 8.90 |  |  |
|  | 0.81 | MUSKARDIA | 103888.65 | -841.55 | 9.19 | 0.30 | -2847.52 |
|  | 1.00 | placebo | 104730.20 |  | 9.00 |  |  |
|  | 1.00 | MUSKARDIA | 103888.65 | -841.55 | 9.27 | 0.27 | -3069.46 |
| u_cda2 | 0.63 | placebo | 104730.20 |  | 8.74 |  |  |
|  | 0.63 | MUSKARDIA | 103888.65 | -841.55 | 9.07 | 0.33 | -2572.54 |
|  | 0.82 | placebo | 104730.20 |  | 9.29 |  |  |
|  | 0.82 | MUSKARDIA | 103888.65 | -841.55 | 9.50 | 0.21 | -4009.89 |
|  | 1.00 | placebo | 104730.20 |  | 9.84 |  |  |
|  | 1.00 | MUSKARDIA | 103888.65 | -841.55 | 9.93 | 0.09 | -9089.05 |
| u_str1 | 0.22 | placebo | 104730.20 |  | 8.71 |  |  |
|  | 0.22 | MUSKARDIA | 103888.65 | -841.55 | 9.05 | 0.34 | -2486.50 |
|  | 0.37 | placebo | 104730.20 |  | 8.75 |  |  |
|  | 0.37 | MUSKARDIA | 103888.65 | -841.55 | 9.08 | 0.33 | -2570.97 |
|  | 0.51 | placebo | 104730.20 |  | 8.80 |  |  |
|  | 0.51 | MUSKARDIA | 103888.65 | -841.55 | 9.11 | 0.32 | -2661.21 |
|  | 0.66 | placebo | 104730.20 |  | 8.84 |  |  |
|  | 0.8 | MUSKARDIA | 103888.65 | -841.55 | 9.17 | 0.34 | -2509.71 |
|  | 0.66 | placebo | 104730.20 |  | 8.84 |  |  |
|  | 0.80 | MUSKARDIA | 103888.65 | -841.55 | 9.17 | 0.34 | -2509.71 |
| u_str2 | 0.35 | placebo | 104730.20 |  | 8.60 |  |  |
|  | 0.35 | MUSKARDIA | 103888.65 | -841.55 | 8.97 | 0.37 | -2268.28 |
|  | 0.63 | placebo | 104730.20 |  | 8.90 |  |  |
|  | 0.63 | MUSKARDIA | 103888.65 | -841.55 | 9.19 | 0.29 | -2919.94 |
|  | 0.90 | placebo | 104730.20 |  | 9.20 |  |  |
|  | 0.90 | MUSKARDIA | 103888.65 | -841.55 | 9.41 | 0.21 | -4096.75 |
| rrcda | 0.32 | placebo | 104730.20 |  | 8.80 |  |  |
|  | 0.32 | MUSKARDIA | 92529.21 | -12201.00 | 9.37 | 0.56 | -21726.97 |
|  | 0.81 | placebo | 104730.20 |  | 8.80 |  |  |
|  | 0.81 | MUSKARDIA | 110093.11 | 5362.91 | 8.98 | 0.18 | 30222.09 |
|  | 1.30 | placebo | 104730.20 |  | 8.80 |  |  |
|  | 1.30 | MUSKARDIA | 120403.91 | 15673.70 | 8.75 | -0.05 | -307146.85 |
| c_qd | 5088.10 | placebo | 104730.20 |  | 8.80 |  |  |
|  | 5088.10 | MUSKARDIA | 101900.72 | -2829.49 | 9.12 | 0.32 | -9011.97 |
|  | 5270.60 | placebo | 104730.20 |  | 8.80 |  |  |
|  | 5270.60 | MUSKARDIA | 103341.25 | -1388.96 | 9.12 | 0.32 | -4423.85 |
|  | 5453.10 | placebo | 104730.20 |  | 8.80 |  |  |
|  | 5453.10 | MUSKARDIA | 104781.78 | 51.57 | 9.12 | 0.32 | 164.26 |
| c_cg | 2036.70 | placebo | 92942.62 |  | 8.80 |  |  |
|  | 2036.70 | MUSKARDIA | 90059.56 | -2883.07 | 9.12 | 0.32 | -9182.62 |
|  | 3788.70 | placebo | 104730.20 |  | 8.80 |  |  |
|  | 3788.70 | MUSKARDIA | 103888.65 | -841.55 | 9.12 | 0.32 | -2680.37 |
|  | 5540.70 | placebo | 116517.79 |  | 8.80 |  |  |
|  | 5540.70 | MUSKARDIA | 117717.74 | 1199.96 | 9.12 | 0.32 | 3821.88 |
| c_str1 | 5631.88 | placebo | 103099.30 |  | 8.80 |  |  |
|  | 5631.88 | MUSKARDIA | 102696.37 | -402.92 | 9.12 | 0.32 | -1283.32 |
|  | 11216.24 | placebo | 104690.59 |  | 8.80 |  |  |
|  | 11216.24 | MUSKARDIA | 103859.69 | -830.90 | 9.12 | 0.32 | -2646.43 |
|  | 16800.60 | placebo | 106281.88 |  | 8.80 |  |  |
|  | 16800.60 | MUSKARDIA | 105023.01 | -1258.88 | 9.12 | 0.32 | -4009.55 |
| c_str2 | 9071.65 | placebo | 101405.65 |  | 8.80 |  |  |
|  | 9071.65 | MUSKARDIA | 101474.49 | 68.85 | 9.12 | 0.32 | 219.28 |
|  | 12095.54 | placebo | 104730.20 |  | 8.80 |  |  |
|  | 12095.54 | MUSKARDIA | 103888.65 | -841.55 | 9.12 | 0.32 | -2680.36 |
|  | 15119.42 | placebo | 108054.75 |  | 8.80 |  |  |
|  | 15119.42 | MUSKARDIA | 106302.80 | -1751.95 | 9.12 | 0.32 | -5580.01 |
| c_cda1 | 16080.16 | placebo | 93973.32 |  | 8.80 |  |  |
|  | 16080.16 | MUSKARDIA | 95307.21 | 1333.90 | 9.12 | 0.32 | 4248.49 |
|  | 26264.94 | placebo | 99712.27 |  | 8.80 |  |  |
|  | 26264.94 | MUSKARDIA | 99885.53 | 173.26 | 9.12 | 0.32 | 551.85 |
|  | 36449.71 | placebo | 105451.22 |  | 8.80 |  |  |
|  | 36449.71 | MUSKARDIA | 104463.84 | -987.37 | 9.12 | 0.32 | -3144.79 |
| c_cda2 | 8944.55 | placebo | 95907.92 |  | 8.80 |  |  |
|  | 8944.55 | MUSKARDIA | 96956.39 | 1048.46 | 9.12 | 0.32 | 3339.36 |
|  | 11926.07 | placebo | 104730.22 |  | 8.80 |  |  |
|  | 11926.07 | MUSKARDIA | 103888.66 | -841.56 | 9.12 | 0.32 | -2680.38 |
|  | 14907.58 | placebo | 113552.51 |  | 8.80 |  |  |
|  | 14907.58 | MUSKARDIA | 110820.94 | -2731.58 | 9.12 | 0.32 | -8700.12 |
| rrstr | 0.31 | placebo | 104730.20 |  | 8.80 |  |  |
|  | 0.31 | MUSKARDIA | 102542.28 | -2187.93 | 9.24 | 0.43 | -5069.58 |
|  | 1.04 | placebo | 104730.20 |  | 8.80 |  |  |
|  | 1.04 | MUSKARDIA | 105009.25 | 279.05 | 9.02 | 0.22 | 1276.00 |
|  | 1.77 | placebo | 104730.20 |  | 8.80 |  |  |
|  | 1.77 | MUSKARDIA | 107260.16 | 2529.96 | 8.84 | 0.03 | 74301.32 |
| p_cdaff | 0.03 | placebo | 103909.49 |  | 8.80 |  |  |
|  | 0.03 | MUSKARDIA | 103244.34 | -665.15 | 9.12 | 0.32 | -2118.53 |
|  | 0.04 | placebo | 104749.56 |  | 8.80 |  |  |
|  | 0.04 | MUSKARDIA | 103903.85 | -845.72 | 9.12 | 0.31 | -2693.62 |
|  | 0.05 | placebo | 105169.60 |  | 8.80 |  |  |
|  | 0.05 | MUSKARDIA | 104563.36 | -606.24 | 9.12 | 0.31 | -1930.89 |
| p_strff | 0.06 | placebo | 104772.69 |  | 8.80 |  |  |
|  | 0.06 | MUSKARDIA | 103919.52 | -853.17 | 9.12 | 0.31 | -2717.37 |
|  | 0.09 | placebo | 104743.60 |  | 8.80 |  |  |
|  | 0.09 | MUSKARDIA | 103898.38 | -845.22 | 9.12 | 0.31 | -2692.03 |
|  | 0.12 | placebo | 104714.50 |  | 8.80 |  |  |
|  | 0.12 | MUSKARDIA | 103877.24 | -837.26 | 9.12 | 0.31 | -2666.69 |
| c_cdadie | 17581.09 | placebo | 103291.22 |  | 8.80 |  |  |
|  | 17581.09 | MUSKARDIA | 102678.77 | -612.44 | 9.12 | 0.31 | -1950.64 |
|  | 23441.46 | placebo | 104730.20 |  | 8.80 |  |  |
|  | 23441.46 | MUSKARDIA | 103888.65 | -841.55 | 9.12 | 0.31 | -2680.37 |
|  | 29301.82 | placebo | 106169.19 |  | 8.80 |  |  |
|  | 29301.82 | MUSKARDIA | 105098.52 | -1070.67 | 9.12 | 0.31 | -3410.09 |
| c_strdie | 21844.82 | placebo | 104269.53 |  | 8.80 |  |  |
|  | 21844.82 | MUSKARDIA | 103554.11 | -715.42 | 9.12 | 0.31 | -2278.62 |
|  | 29126.43 | placebo | 104730.20 |  | 8.80 |  |  |
|  | 29126.43 | MUSKARDIA | 103888.65 | -841.55 | 9.12 | 0.31 | -2680.37 |
|  | 36408.03 | placebo | 105190.88 |  | 8.80 |  |  |
|  | 36408.03 | MUSKARDIA | 104223.18 | -967.69 | 9.12 | 0.31 | -3082.11 |
| cycle | 10.00 | placebo | 48461.93 |  | 5.98 |  |  |
|  | 10.00 | MUSKARDIA | 51505.12 | 3043.18 | 6.11 | 0.13 | 23616.20 |
|  | 15.00 | placebo | 71247.03 |  | 7.43 |  |  |
|  | 15.00 | MUSKARDIA | 72401.89 | 1154.86 | 7.65 | 0.22 | 5274.53 |
|  | 20.00 | placebo | 87677.44 |  | 8.22 |  |  |
|  | 20.00 | MUSKARDIA | 87710.72 | 33.28 | 8.50 | 0.28 | 120.83 |
|  | 25.00 | placebo | 98081.39 |  | 8.61 |  |  |
|  | 25.00 | MUSKARDIA | 97559.29 | -522.10 | 8.91 | 0.30 | -1725.45 |
|  | 30.00 | placebo | 104730.20 |  | 8.80 |  |  |
|  | 30.00 | MUSKARDIA | 103888.65 | -841.55 | 9.12 | 0.32 | -2680.37 |

Note: Placebo group: placebo combined with standard treatment; MUSKARDIA group: MUSKARDIA combined with standard treatment；rate: discount rate; rrcda: RR of non-fatal MI; rrstr: RR of non-fatal stroke; u_cda1: utility of non-fatal MI; u_cda2: utility of post-MI; u_str1: utility of non-fatal stroke; u_str2: utility of post-stroke; c_cda1: cost of non-fatal MI; c_cda2: cost of post-MI; c_str1: cost of non-fatal stroke; c_str2: cost of post-stroke; c_qd: Annual cost of Shexiang Baoxin Pill; c_cg: Annual cost of standard therapy; c_cdadie: cost of death due to MI; c_strdie: cost of death due to stroke; p_cdaff: MI recurrence rate; p_strff: stroke recurrence rate.
